# Argument against the Routine Use of Steroids for Pediatric Acute Respiratory Distress Syndrome

**DOI:** 10.3389/fped.2016.00079

**Published:** 2016-07-28

**Authors:** Silvia M. Hartmann, Catherine L. Hough

**Affiliations:** ^1^Pediatric Critical Care Medicine, Seattle Children’s Hospital, University of Washington School of Medicine, Seattle, WA, USA; ^2^Pulmonary and Critical Care Medicine, Harborview Medical Center, University of Washington School of Medicine, Seattle, WA, USA

**Keywords:** pediatric ARDS, steroids, mortality, ventilator-free days, heterogeneity

## Abstract

Steroids have a plausible mechanism of action of reducing severity of lung disease in acute respiratory distress syndrome (ARDS) but have failed to show consistent benefits in patient-centered outcomes. Many studies have confounding from the likely presence of ventilator-induced lung injury and steroids may have shown benefit because administration minimized ongoing inflammation incited by injurious ventilator settings. If steroids have benefit, it is likely for specific populations that fall within the heterogeneous diagnosis of ARDS. Those pediatric patients with concurrent active asthma or reactive airway disease of prematurity, in addition to ARDS, are the most common group likely to derive benefit from steroids, but are poorly studied. With the information currently available, it does not appear that the typical adult or pediatric patient with ARDS derives benefit from steroids and steroids should not be given on a routine basis.

## Introduction

It is widely accepted among intensivists that dysregulated inflammation in response to an inciting infection or injury is a key pathophysiologic feature of acute respiratory distress syndrome (ARDS). Glucocorticoids have known effects in dampening many aspects of the immune and inflammatory response and, therefore, it is biologically plausible that steroids would minimize disease severity in the patient with ARDS. Although the administration of corticosteroids for anti-inflammatory effect is an intuitive, widely available, and inexpensive therapy for ARDS, the receipt of steroids has not decreased mortality or improved other patient-centered outcomes. Despite a plausible mechanism of action, steroid treatment has also failed to show consistent benefit in other inflammatory disease processes, such as sepsis (1), indicating a fundamental challenge in the appropriate use of this class of medications for inflammatory disorders. The challenge remains poorly characterized with many hypotheses as to why steroids have not provided benefit for ARDS. Perhaps steroids are administered too late in the inflammatory process where redundant mechanisms of activation allow for ongoing pulmonary damage. Perhaps the paths to ARDS are significantly heterogeneous such that some subgroups would benefit from steroids, while others experience harm, and studying these groups together blurs the treatment effects.

Although the cumulative literature cannot endorse the routine use of steroids for ARDS, they are frequently prescribed – ~40% of patients received corticosteroids in several large trials (2, 3). Perhaps steroids remain in the intensivists’ armamentarium for ARDS because there are currently few alternative therapies to minimize pulmonary inflammation and maladaptive fibrotic repair mechanisms. A better understanding of how patients’ lungs are able to control inflammation and recreate functional tissue will likely offer avenues of therapy in future years.

In general, there are three indications for which steroids may be considered for the patient with ARDS: (1) treatment of a coexistent steroid-responsive disease, (2) treatment of inflammation early in the course of ARDS to minimize lung damage, and (3) treatment of maladaptive healing responses late in the course of ARDS to improve lung function. Studies involving each group are considered separately and discussed below. Pediatric data are highlighted when available. Lastly, adult studies exploring the possible association of steroids with neuromuscular weakness are reviewed as a cautionary note that we may not understand all of the risks associated with steroid therapy. No similar pediatric data exist. Currently, practitioners have to decide if the potential benefits of steroids to treat ARDS outweigh possible risks of both short- and long-term morbidities using incomplete data. In most cases of ARDS, steroid therapy is unlikely to provide improved patient outcomes.

## Treatment of Co-Existing Steroid-Responsive Diseases

Steroids may be prescribed to patients with ARDS to treat chronic pulmonary disorders that complicate the treatment of lung disease. This is a poorly studied group of patients but with a strong physiologic rationale for steroid treatment. Steroids alternatively may be prescribed to patients with ARDS with an acute process thought to be independently treatable with glucocorticoid therapy. This group of disorders includes diagnoses, such as community-acquired pneumonia (CAP), some etiologies of diffuse alveolar hemorrhage, and *Pneumocystis jirovecii* pneumonia. Only CAP will be discussed in detail as it is the most commonly encountered diagnosis and is a key risk factor for ARDS.

It is likely that there is a notable minority of patients with ARDS who have underlying chronic pulmonary diseases that are routinely treated with steroids. These include the familiar examples of chronic obstructive pulmonary disease (COPD) or asthma in adults and reactive airway disease and chronic lung disease of prematurity in the pediatric population. Based on reported numbers of ARDS patients who met inclusion criteria for large randomized controlled trials, ~9–15% were excluded for “chronic pulmonary disease” (4, 5), some of whom likely have diagnoses that respond positively to steroid therapy. A single institution prospective cohort of patients with moderate-to-severe pediatric ARDS (PARDS) noted that 15% had reactive airway disease and most of these patients received steroids (6). Based on these numbers, it is relatively common to have patients with ARDS who may have an active co-existing steroid-responsive disease.

Although the cohort with exacerbations of obstructive lung disease who meet consensus definitions of ARDS/PARDS are the most logical group to treat with glucocorticoids, this population is poorly studied. Many of the large randomized controlled trials in adults that have shaped the way ARDS is treated today have excluded patients with “severe chronic respiratory disease” (4, 7–9). This group is captured as those with pre-existing hypercarbia, spirometry consistent with obstructive lung disease, home supplemental oxygen or non-invasive positive pressure ventilation, radiographic changes of obstructive lung disease, or some combination of these criteria. Other studies have excluded this cohort by excluding concurrent beta-2 agonist therapy (10). These definitions likely exclude some, but not all, patients with underlying steroid-responsive diseases. As such, the current ARDS literature does not help evaluate the spectrum of patients who may be experiencing concurrent steroid-responsive obstructive lung disease while hospitalized with ARDS. Similarly, in the pediatric literature patients with potentially steroid-responsive chronic disorders have been excluded in some studies and included in others without reporting the presence of these diagnoses. For example, a study of surfactant for PARDS excluded children with status asthmaticus and chronic lung disease defined as use of home supplemental oxygen or diuretic medications (11). Another study of prone positioning did not specifically exclude children with potential steroid-responsive diseases, but did not report whether these patients were enrolled (12).

Adults receiving steroids for other underlying diseases, including recent asthma exacerbation, have been appropriately excluded from trials specifically for the evaluation of steroids in ARDS (5). A feasibility trial of steroids for PARDS included patients with asthma or potential prematurity-associated chronic lung disease and they comprised 20% of a small cohort (13), which may skew the results in a favorable direction for the use of steroids in PARDS. Any future investigation of steroids to treat ARDS should analyze patients with steroid-responsive chronic lung disease separately, as it is a challenge to understand the degree of impact steroids have on the obstructive vs. restrictive lung disease components of illness. These patients with active asthma, COPD, or prematurity-associated chronic lung disease complicating ARDS have the strongest rationale to have a positive response to routine steroid administration and may have the most optimal risk-benefit ratio among the three general indications for steroids.

Steroid administration has also been investigated to treat acute pulmonary disorders that are risk factors for ARDS. Two recent meta-analyses have reviewed randomized controlled trials and cohort studies that both concluded that there is a lower risk of ARDS when low-moderate doses of steroids are given in patients with CAP (14, 15). One review noted that average dosing of steroids for treatment of CAP to be lower than doses used to treat both early and late ARDS in many studies (15). Although there is a positive relationship between steroids and CAP, the finding does not indicate that steroids should be effective for patients with ARDS secondary to CAP, as this patient population is different by virtue of experiencing more robust pulmonary inflammation. There may be a “window of opportunity” in which steroids may benefit CAP to prevent progression to ARDS, but steroids probably are not useful once a patient has significant enough disease to meet ARDS criteria. The difference in the literature of steroid treatment for CAP vs. steroid treatment for ARDS highlights the importance of timing of steroid therapy. Many patients with ARDS likely present to medical attention beyond a potential “window of opportunity” for positive steroid effect.

In summary, there is a high likelihood that steroid administration to patients with ARDS/PARDS with coexistent and active asthma, reactive airway disease associated with prematurity, or COPD will be beneficial based on the known pathophysiologic mechanisms of obstructive lung disorders. However, there is a paucity of clinical evidence to support this hypothesis. It is unlikely that steroids to treat acute pneumonia will improve outcome once patients have met criteria for ARDS as the timing of steroid administration is too late to mitigate the severity of inflammation.

## Treatment of Early ARDS to Minimize Pulmonary Inflammation

Administration of steroids within the first 72 h of mechanical ventilation for ARDS aims to dampen the exuberant inflammatory response and translate into milder pulmonary disease, marked by an increase in ventilator-free days and lower mortality. The majority of data for treatment of early ARDS with steroids were gathered when ventilator strategies likely incurred significant lung injury that impacted both clinical outcomes of mortality and ventilator-free days. It is unclear whether steroids in this setting positively affected patient outcome by minimizing the ongoing inflammation produced by the ventilator. There is very limited information about steroid administration in patients receiving lung protective ventilation.

A variety of steroid dosing regimens have been studied. Initially, high doses of methylprednisolone (30 mg intravenously every 6 h) to treat early ARDS were investigated but there was no mortality benefit (16) nor was there evidence that steroid administration could prevent ARDS in high risk populations (17–20). More recent investigations have focused on low–moderate doses of steroids with modest variability in the exact dosing regimen.

Investigation of low–moderate doses of steroids to treat early ARDS gained enthusiasm after a *post hoc* subgroup of patients included in a trial of hydrocortisone and fludrocortisone treatment for septic shock (21). This group was somewhat more homogeneous than other studies as all patients included had secondary ARDS in the setting of sepsis. Approximately 14% of this subgroup also had chronic pulmonary diseases. The administration of 50 mg of hydrocortisone intravenously every 6 h for 7 days in conjunction with 50 μg of fludrocortisone daily was not associated with any mortality benefit for the group as a whole. However, the subgroup of patients who were diagnosed with critical-illness related adrenal insufficiency (CIRCI), also denoted as non-responders, had a significant difference in mortality with the administration of steroids (53 vs. 75%, 95% CI: 0.36–0.89, *p* = 0.013) compared to those who received placebo. There were approximately equal numbers of patients who had chronic pulmonary disorders in the non-responder placebo and non-responder steroid groups. Some have hypothesized that ARDS is the pulmonary manifestation of inappropriately low endogenous cortisol production for an inflammatory stimulus (22). Given that ARDS is a heterogeneous group of patients, there may be certain subgroups, such as patients with CIRCI, that may benefit from steroids administration for ARDS but that conclusion cannot be made from a *post hoc* analysis of relatively small numbers (*N* = 177) of patients who did not receive the lung-protective ventilation that is now standard of care.

The most positive study results in favor of treating ARDS with steroids is from a small randomized controlled trial of 91 patients conducted by Meduri and colleagues specifically to investigate the treatment of low–moderate doses of methylprednisolone for the treatment of early ARDS (23). This study has significant limitations that are worth discussing in detail. Patients were recruited between 1997 and 2002. This study participants appeared to be a relatively severely ill group of patients with average positive end-expiratory pressure of 12 cm of H_2_O, PaO_2_/FiO_2_ ratio of about 120, and lung injury score (LIS) of ~3 upon enrollment. The trial compared a regimen of methylprednisolone to placebo. The methylprednisolone regimen included a 1 mg/kg loading dose followed by a 1 mg/kg/day infusion that was planned for 14 days with a taper for an additional 14 days. Steroids could be decreased more rapidly if patients met the study outcome of successful extubation or a decrease in LIS by 1 by day 7 of treatment. Patients were randomized in a 2:1 fashion such that 63 patients were in the steroid arm and 28 were in the control group. This study showed significantly positive results in all outcome measures. Almost 70% of the steroid group had a one-point decrease in LIS by day 7 of the study compared to 36% of the control group (*p* = 0.002). Approximately 54% of patients in the steroid group extubated successfully by day 7 compared to 25% of control patients (*p* = 0.01). Mortality was also lower in the steroid group (24 vs. 43%, *p* = 0.07). The results of this trial support ongoing investigation into steroids to treat certain patient populations with ARDS but the limitations of the study preclude treating *all* patients with ARDS with routine steroid therapy. For example, the methodology used by the authors initially limited plateau pressures to 35 cm of H_2_O and was later changed to 30 cm of H_2_O in accordance with the ARDSnet publication (7). The actual plateau pressures or tidal volumes received by patients are not reported and, thus, introduce potential confounding by ventilator-induced lung injury. The overall small number of participants and especially the small number of control patients, the possible participation of patients with steroid-responsive chronic conditions, the outcome measure of extubation rather than ventilator-free days, and the lack of utility of LIS as an outcome measure (24) could all be addressed in a future trial.

The results of a recent pilot trial cast doubt on the positive findings of the Meduri study. Although the cohorts evaluated by these two studies are not equivalent, this newer data bring equipoise to the question of steroids for ARDS. This small study was conducted in South Korea to determine predictive factors for response to steroids in patients with moderate-to-severe ARDS (25). The study involved 20 patients with mostly moderate ARDS by the Berlin criteria. This cohort has a lower severity of illness compared to the study by Meduri. The group reported using ARDSnet protocol for ventilation but does not report the ventilator parameters that participants actually received. All of these participants received a methylprednisolone loading dose and infusion for the first 14 days of the study that is comparable to the dosing used in the Meduri study. The study used the same outcome measures of extubation or a one-point decrease in LIS by day 7 of steroid therapy to define a group of “responders” and “non-responders.” Fifty percent of the cohort was deemed to have a response to steroid therapy. The most interesting part of this study is that the authors tried to predict the group of responders with biomarkers from serum and bronchoalveolar lavage (BAL) specimens that were collected at enrollment and on study day 7. Biomarkers collected included serum procalcitonin, C-reactive protein (CRP), transforming growth factor-beta (TGF-β), interleukin-6 (IL-6), tumor necrosis factor-alpha (TNF-α), *N*-terminal pro-B type natriuretic peptide (NT-proBNP). Biomarkers from BAL samples included triggering receptor expressed on myeloid cells (TREM-1), which has been associated with bacterial infection, and procollagen peptide type III, which is thought to represent ongoing fibroproliferation in the lung. Previous studies performed before lung protective ventilation demonstrated higher inflammatory cytokines in BAL samples that correlated to the ongoing need for mechanical ventilation that was presumed to be from fibroproliferation and also correlated with mortality (26, 27). However, in this study, there was no significant difference in any of the inflammatory markers or procollagen peptide III between responders and non-responders. Nor did the investigators find a difference in clinical metrics, such as initial oxygenation index, PaO_2_/FiO_2_ ratio, or LIS. The response rate to steroids was surprisingly low and it is unclear whether the poor response rate indicates that fewer patients may benefit from steroids than previously hypothesized, or whether the investigators identified differences in the “natural history” of pulmonary healing between the two different groups. Regardless, it is important to acknowledge that despite using the best available clinical parameters and biomarkers of disease severity, we yet cannot identify individuals for the directed use of steroid as personalized therapy or for enrollment of an enriched cohort of likely steroid responders into future clinical trials.

There has been less investigation into the possible role of steroids in PARDS. A pilot trial enrolled 35 pediatric patients with an average PaO_2_/FiO_2_ ratio of ~150 and compared duration of mechanical ventilation in a group receiving steroid to one receiving placebo (13). The intervention group received a different steroid regimen than the adult trials. A loading dose of 2 mg/kg of intravenous methylprednisolone was followed by a 1 mg/kg methylprednisolone infusion for 7 days and then a taper between 7 and 14 days. This represents a somewhat shorter course of steroids compared to previous adult studies. Both groups had a small, but equal number of patients who may potentially have steroid-responsive diseases, including asthmatics and preterm infants. The intervention arm had on average lower lung compliance for the first two study days with higher plateau pressures but equivalent tidal volumes (average plateau pressure 33 cm H_2_O vs. 24 cm H_2_O, *p* = 0.006). There was no difference in the duration of mechanical ventilation between the groups. The sample size is too small to determine if the steroids improved lung compliance that enabled the intervention group to extubate at generally the same time as the control group, despite what might be more significant lung disease.

In contrast to the somewhat optimist results of the previously described pediatric feasibility study of methylprednisolone, a larger prospective observational cohort pointed toward steroid therapy being associated with fewer ventilator-free days and increased mortality (6). A group of 283 children who met both adult and pediatric definitions of moderate-to-severe ARDS were followed throughout their hospital course. Sixty percent of the cohort was exposed to steroids for more than 24 h, a criterion meant to exclude patients receiving peri-extubation steroids and brief courses of “stress dose” steroids. Of the patients who received more than 24 h of steroids, 22% of them had a history of reactive airway disease. Steroids were initiated on the same day as PARDS diagnosis in most patients (IQR 0–1) with a median duration of 7 days (IQR 5–12 days). Patients received different corticosteroids with the most common being hydrocortisone (51%) and methylprednisolone (41%). Steroid dose was compared using methylprednisolone equivalents with a median cumulative dose of 8 mg/kg (IQR 4–20 mg/kg) during mechanical ventilation. The average patient in this study received ~1 mg/kg of corticosteroids, which is similar to the low–moderate dosing in adult studies. Steroid exposure greater than 24 h was independently associated with fewer ventilator-free days when adjusted for severity of illness, immunocompromised status, number of non-pulmonary organ failures, reactive airway disease, and worst oxygenation index in the first 24 h, maximum vasopressor score in the first 72 h, and fluid balance in the first 72 h of PARDS. Surprisingly, the mortality was higher in the group receiving steroids for greater than 24 h compared to the group with no steroid/steroid for less than 24 h (17 vs. 8%, *p* = 0.034, unadjusted analysis). Cumulative steroid dose was also associated with increased mortality among patient with PARDS. This study has several limitations. The variability of the steroid regimens, including the specific corticosteroid, the dose, and how the medication was discontinued may influence outcomes. Steroids may have been used for different indications in patients that are not readily apparent in the reported data. Importantly, the cohort design cannot control for unknown confounders with statistical analysis. Despite these limitations, the difference in mortality that is adjusted for severity of illness should make the clinician cautious with the use of steroids for PARDS until more robust information is available.

The literature for both pediatric and adult patients with ARDS in its current state cannot support routine steroid use within the first 72 h. The studies with positive results should encourage the medical community to continue to investigate which subgroups may benefit from steroids in addition to lung protective ventilation, e.g., patients with *P. jirovecii* pneumonia (28, 29), well as those groups who may suffer additional harm, e.g., patients with H_1_N_1_ pneumonia (30–32).

## Treatment of Late ARDS to Inhibit Fibroproliferation

During the early phase of ARDS, there is simultaneous activation of inflammation, coagulation, and tissue repair mechanisms. Over time, some survivors control the level of inflammation and repair the pulmonary tissue, returning it to a good level of function. On the other hand, some survivors have persistent inflammation and a maladaptive repair process involving the deposition of extracellular matrix that leads to ongoing restrictive lung disease. Studies have looked at the ability of steroids, which are known to inhibit the fibrotic response in addition to anti-inflammatory effects, to improve lung function when patients have failed to extubate by day 7 of ARDS with the assumption that this group has significant pulmonary fibroproliferation and ongoing inflammation. This assumption is supported by a recent publication from a single center that routinely obtains open lung biopsy in patients with unresolving ARDS that can clinically tolerate the procedure (33). The study included 83 adult patients with variable severity of disease. The median time to lung biopsy was 8 days. Overall, 58% of patients with ARDS still on the ventilator at the time of lung biopsy had evidence of ongoing diffuse alveolar damage and fibroproliferation. These histologic findings were more common in patients with severe ARDS as defined by the Berlin criteria. Steroid use was not reported.

In 1998, Meduri and colleagues published the results of a small randomized controlled trial that supported the use of steroids for late ARDS (34). They randomized 24 adults with ongoing ventilator need 7 days after ARDS diagnosis and with little improvement in LIS in a 2:1 fashion to methylprednisolone treatment vs. placebo. The dosing of methylprednisolone was higher than the dosing used in many of the early ARDS studies. Patients received 2 mg/kg of intravenous methylprednisolone as a loading dose and continued on intermittent dosing every 6 h for a total of 2 mg/kg/day for 14 days. The steroid was tapered over 18 days for a total of 32 days of steroid therapy. The protocol allowed for cross-over from the placebo group into the steroid group if there was no improvement in LIS or extubation by study day 10. The primary outcome was mortality. The study was stopped early for significantly better survival in the steroid group – all patients in the steroid arm survived and 5 of 8 (60%) of the placebo arm died. The study is statistically challenging to interpret with a very small control group of only 8. The study has been criticized for a high cross-over rate of 50% from the placebo into the steroid arm with a significant number of deaths in the placebo group occurring after receiving steroids. Additionally, similar to many studies for early ARDS, these patients did not receive standard lung-protective ventilation leading to the hypothesis that steroids may have been treating the ongoing inflammation incurred by ventilator-induced lung injury.

A larger randomized controlled trial from the ARDSnet investigators followed the initial small trial with results that did not support routine administration of steroids for ARDS (5). This trial enrolled 180 adults with a diagnosis of ARDS that had persistent pulmonary infiltrates and ongoing mechanical ventilation for 7 days. The study failed to recruit the estimated sample size of 400 despite enrolling patients at 25 hospitals. The protocol for methylprednisolone was modified from the Meduri study to allow a faster taper over 4 days if extubation occurred, but was otherwise relatively similar. Mortality was equivalent in both groups. The steroid arm had significantly fewer days of mechanical ventilation (~7 days vs. 11 days, *p* < 0.001). However there was a significantly higher need for reinstitution of mechanical ventilation in the steroid group (20 vs. 6, *p* = 0.008). Hypotheses to explain a higher reintubation rate include recrudescence of pulmonary inflammation in the setting of a rapid steroid taper, steroid-associated neuromyopathy, or undiagnosed infection. Of note, there was an *a priori* subgroup analysis of patients who enrolled after 13 days of ARDS. This cohort is small with 23 patients in the steroid group and 25 in the placebo group. Similar to the study by Meduri, these patients had an overall higher mortality (44 vs. 12%). The authors concluded that steroid administration after 7 days does not have clear benefit and administration of steroids after 14 days may be harmful. If steroids are useful for fibroproliferation, there may be a window of opportunity for administration where fibroproliferation may be modified.

In summary, the data from adult patients with late ARDS and presumed ongoing fibroproliferation do not show mortality benefit. Furthermore, steroids have only a questionable benefit in decreasing morbidity; despite decreasing the overall duration of mechanical ventilation. There is an associated high rate of reintubation and no overall decrease in length of hospital stay with the currently studied doses and regimens. Specifically, steroids should not be used as standard of care for pediatric patients who still have ongoing need for mechanical ventilation after a week based on the adult data. An additional layer of uncertainty in younger children with ongoing lung development is whether there are similar rates of fibroproliferation and ongoing diffuse alveolar damage that are the conceptual basis for steroid therapy.

## Possible Long-Term Effects of Steroid Treatment for ARDS

As survival has improved for disorders treated in the intensive care unit, focus has broadened from hospital markers of good outcome to patient function in society several years after discharge. Steroids given to treat ARDS have been evaluated for short-term pulmonary benefits and mortality in the studies reviewed above, but steroid use has also been implicated in ICU-acquired neuromyopathy contributing to physical impairments seen in survivors of ARDS that lasts months if not years, after hospital discharge (3, 35–37). Confirming the causality of steroid therapy in the development of ICU-acquired neuromyopathy is challenging as most studies cannot control for other contributing factors to ongoing weakness, such as the use of neuromuscular blockade and general immobility while ventilated. Although it is impossible to use the current information to help guide the decision to give or withhold steroids during early or late ARDS, providers should be aware of the controversy around ICU-acquired neuromyopathy and understand that other currently unrecognized long-term consequences of ICU therapy, including steroids, may arise over time.

ICU-acquired neuromuscular weakness involves dysfunction of the nerves, muscles, or both. Nerves may be functionally impaired or completely denervated. Skeletal muscles can have both impaired contractility and diminished mass from a combination of decreased protein synthesis and increased proteolysis. On the one hand, steroids may have direct myotoxicity but, on the other hand, steroids may decrease the duration of mechanical ventilation and minimize the contribution of bedrest to ICU-acquired weakness. One study of 95 mechanically ventilated patients found an odds ratio of nearly 15 (95% CI: 3.2–69.8) for development of ICU-acquired neuromyopathy if patients had received steroids (38). However, these patients were also sedated to the point where they could not follow commands for an average of 2 weeks. It is unclear whether it is the receipt of corticosteroids or immobilization that is the more important factor in the severity of ICU-acquired neuromuscular weakness.

There are two studies that followed patients for a year after hospital discharge that found an association between steroid receipt and ongoing weakness that support the hypothesis of direct myotoxicity from steroids. Herridge and colleagues followed a cohort of patients with moderate-to-severe ARDS for 12 months (35). This group of 109 patients was a relatively sick cohort with a median duration of mechanical ventilation of 21 days that were likely not treated with lung protective ventilation as the hospitalizations were between 1998 and 2001. This study found the absence of corticosteroid treatment the most predictive factor in improvement in 6-min walk test during the first 3 months after hospital discharge and the strength of this association decreased and disappeared over time as might be expected from a slowly reversible process. There is a strong possibility of residual confounding with the sickest patients that are included in this study receiving steroids and then noting worse outcomes in an observational cohort. More recently, a cohort of patients from various ARDSnet trials were followed at 6 and 12 months after hospital discharge (3). The study consisted of 203 patients, most of whom met definitions of moderate-to severe ARDS within 72 h of admission to the ICU. All patients received lung protective ventilation but steroid administration, neuromuscular blockade, and early mobility were not a part of the study protocols. Forty-three percent of the cohort received steroids with an average daily dose of 52 mg of prednisone equivalents (SD ± 81 mg) and mainly used to treat early ARDS (83% received steroids within 72 h of mechanical ventilation). Most patients (75%) did not receive early mobilization and likely experienced a significant degree of immobility. The study reported that corticosteroids, up to a dose of 40 mg/day, were associated with increasingly worse physical outcome measures but is limited by residual confounding.

Not all follow-up studies of ARDS patients have found an association between ICU-acquired weakness and steroids. One recently published study of 222 adults with ARDS reported no association between cumulative steroid dose and muscle weakness at hospital discharge but rather reported that muscle strength was diminished by 3–11% for each day of bedrest when adjusting for risk factors (36). This study highlights the role that mobilization of patients may have in offsetting neuromuscular weakness, such that interventions that decrease the duration of mechanical ventilation, even steroids, may preserve muscle strength after ARDS. Importantly, a review of 128 survivors from the ARDSnet trial of steroids for late ARDS used data from a randomized controlled trial that controlled for other confounding variables (37). Although the diagnosis of neuromyopathy was significantly more common in the group randomized to steroids in the ARDSnet study (5), there was no significant difference between clinician recognition of neuromyopathy during the hospitalization between the two groups in the later review of survivors (37).

The pediatric literature is very limited in describing long-term outcomes for patients with PARDS (39). Children have persistent pulmonary impairment in several case series summarized by the recent consensus conference on PARDS, but neurocognitive outcomes and ability to return to school remain research priorities. The adult studies of corticosteroid administration contributing to ongoing muscle weakness may not capture all of the potential risks of corticosteroids in the youngest patients with ongoing lung and neurocognitive development.

## Conclusion

The summative evidence for steroid administration to the patient with ARDS or PARDS falls short of the level needed to support routine use for either early or late disease. The patients who meet the consensus definitions for ARDS and PARDS continue to be a heterogeneous group with subtle differences in pathophysiology. The positive signal for steroids in some studies combined with higher likelihood of benefit in some subgroups, such as those with steroid-responsive underlying disorders, supports ongoing research into the appropriate use of steroids for ARDS. Perhaps the use of genomics, proteomics, and metabolomics will help identify both cohorts with common diagnoses, as well as individuals, who will benefit from steroid therapy.

Previous studies of corticosteroid therapy for patients with ARDS cannot be directly applied to the care of patients today. Various meta-analyses have been published relatively recently on the use of steroids for ARDS (30, 40–43). All are problematic to interpret because studies of steroids for early and late ARDS have been evaluated together although the therapeutic targets and patient populations are different. No similar meta-analyses exist for steroid therapy in PARDS. Additionally, no single sizable study has a complete cohort of participants who receive lung-protective ventilation and ventilator discontinuation by a strict protocol to reduce the potential confounding from ventilator-induced lung injury. These components of study design are even more important for pediatric studies of PARDS where mortality is unlikely to be affected by an adjunctive respiratory therapy and where ventilator-free days would be a more appropriate short-term outcome. Studies of steroids in adults with ARDS are likely to be generalizable to many older pediatric patients with PARDS after reaching lung maturity, especially teenage patients.

The Pediatric Acute Lung Injury Consensus Conference (PALICC) agreed that steroids cannot be recommended as part of routine care for PARDS (44). While the door is not fully closed on steroid therapy for pediatric and adult ARDS, rational administration of glucocorticoids for this diagnosis requires a reasonable belief that the patient falls in a special cohort of steroid responders. The best-defined steroid responders are those with ARDS from *Pneumocystis pneumonia*. The next most likely group of steroid responders are patients with underlying steroid-responsive obstructive lung disease who have biologic reason to respond to steroids but have been inadequately studied in the setting of concurrent ARDS. These groups together compromise a minority of all patients with ARDS/PARDS and steroid administration should be the exception rather than the rule.

## Author Contributions

SH drafted and revised the editorial. CH critically reviewed the editorial for important intellectual content.

## Conflict of Interest Statement

The authors declare that the research was conducted in the absence of any commercial or financial relationships that could be construed as a potential conflict of interest.
